# Direct observation of genomic heterogeneity through local haplotyping analysis

**DOI:** 10.1186/1471-2164-15-418

**Published:** 2014-06-02

**Authors:** Kamalakar Gulukota, Donald L Helseth Jr, Janardan D Khandekar

**Affiliations:** Center for Molecular Medicine, NorthShore University HealthSystem, 2650 Ridge Ave, Evanston, IL 60201 USA

## Abstract

**Background:**

It has been an abiding belief among geneticists that multicellular organisms’ genomes can be analyzed under the assumption that a single individual has a uniform genome in all its cells. Despite some evidence to the contrary, this belief has been used as an axiomatic assumption in most genome analysis software packages. In this paper we present observations in human whole genome data, human whole exome data and in mouse whole genome data to challenge this assumption. We show that heterogeneity is in fact ubiquitous and readily observable in ordinary Next Generation Sequencing (NGS) data.

**Results:**

Starting with the assumption that a single NGS read (or read pair) must come from one haplotype, we built a procedure for directly observing haplotypes at a local level by examining 2 or 3 adjacent single nucleotide polymorphisms (SNPs) which are close enough on the genome to be spanned by individual reads. We applied this procedure to NGS data from three different sources: whole genome of a Central European trio from the 1000 genomes project, whole genome data from laboratory-bred strains of mouse, and whole exome data from a set of patients of head and neck tumors. Thousands of loci were found in each genome where reads spanning 2 or 3 SNPs displayed more than two haplotypes, indicating that the locus is heterogeneous. We show that such loci are ubiquitous in the genome and cannot be explained by segmental duplications. We explain them on the basis of cellular heterogeneity at the genomic level. Such heterogeneous loci were found in all normal and tumor genomes examined.

**Conclusions:**

Our results highlight the need for new methods to analyze genomic variation because existing ones do not systematically consider local haplotypes. Identification of cancer somatic mutations is complicated because of tumor heterogeneity. It is further complicated if, as we show, normal tissues are also heterogeneous. Methods for biomarker discovery must consider contextual haplotype information rather than just whether a variant “is present”.

**Electronic supplementary material:**

The online version of this article (doi:10.1186/1471-2164-15-418) contains supplementary material, which is available to authorized users.

## Background

In cancer biology, it is well established that histological, ploidy and genomic heterogeneity can occur within different regions of a single tumor [1, 2]. Such cellular diversity is generally assumed to be characteristic of (or caused by) tumor pathology [3]. However, recent reports of genome mosaicism [4] in humans have raised the possibility that such heterogeneity is physiological and can occur without any pathology. Here we report that such cellular heterogeneity at the genomic level is ubiquitous. We introduce the technique of Local Haplotyping Analysis (LHA) which shows that evidence for heterogeneity is strong and directly observable in Next Generation Sequencing (NGS) data.

Single nucleotide polymorphisms (SNPs) are typically deduced from NGS data using a statistical framework which examines the genome site by site [5]. For example, of the NGS reads mapped to a particular position, if half the reads show a C and the other half show a T, a SNP may be “called” at this position. Software packages that implement such SNP-calling procedures like SAMtools [6] and the GATK [7] generally assume a uniform diploid genome. Therefore in this example, a C/T heterozygous SNP would be called.

Mathematically speaking, an alternative explanation is also consistent with the data. Instead of having a C/T heterozygous SNP uniformly, the sequenced tissue might be heterogeneous and consist of two different cell lineages: one of which is homozygous for C and the other homozygous for T. Direct evidence to support such an alternate hypothesis cannot be found when examining a single genomic site. Instead, combinations of sites must be examined and haplotypes must be deduced. However, published methods for haplotype assembly [8] also assume a uniform diploid genome and simply attempt to identify the two most likely haplotypes. In this paper, we break the uniformity assumption. Instead, we examine *all* possible haplotypes with the explicit aim of evaluating evidence for heterogeneity in the tissue.

**Figure 1:**
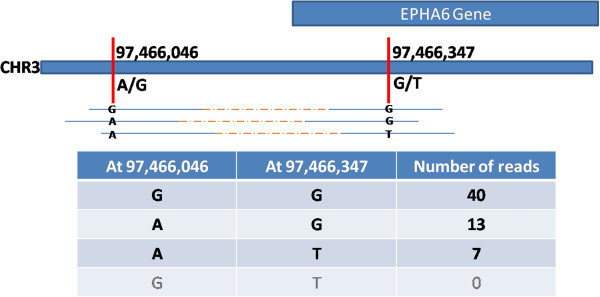
**Cartoon of a 2-SNP block.** Overlapping with the gene EPHA6, we called 2 SNPs on chromosome 3 about 300 bases apart in the CEU_TRIO member NA12878. Since both SNPs are heterozygous and they are within 500 bases of each other, this is a block on the genome. Three example reads are shown along with the bases they map to each SNP position. The table below shows frequencies. For example, there are 40 examples of reads that have a G at each SNP position.

In Figure 1, two sites on chromosome 3 are predicted to have A/G and G/T heterozygosity, respectively. This region can possibly show four sequences or *haplotypes viz. A*..*G*, *A*..*T*, *G*..*G* and *G*..*T* (where the ‘..’ represents the sequence between the SNPs – which is common to all four), based on what combinations of bases are found on a DNA strand. If individual reads span both SNPs, then these combinations are directly observable and it is possible to list haplotypes. Furthermore, if the underlying genome is uniform and diploid, we should only see two of the four possible haplotypes. Seeing three or more constitutes evidence that multiple cell lineages are present in the tissue i.e. that the tissue is heterogeneous.

When the two heterozygous SNPs are far apart from each other, as is usually the case, there are no NGS reads that span both and hence haplotypes are not observable. However, when they are close enough they may be so spanned by reads (or read-pairs in the case of paired-end sequencing) and haplotypes may be directly observable. A single read (pair) can, by definition, be derived only from a single haplotype out of a possible heterogeneous mix. Therefore, listing what each read (pair) shows while it spans neighboring SNPs, is a way to enumerate haplotypes that are directly observed. A region of the genome where two or more SNPs are close enough that a single read (pair) might span it is called a *Block*[8]. Our Local Haplotype Analysis (LHA) pipeline lists reads mapped to blocks to see if there is evidence for more than two haplotypes i.e. for the proposition that the tissue sequenced is a heterogeneous mix of genetically diverse cells.

### Overall strategy of LHA

The starting point of the LHA pipeline is the list of SNPs called from any sequenced genome or exome. SNP calling procedures map the sequenced genome to a reference and examine positions of variation from the reference. Then they routinely apply filters to minimize calling a SNP based on variations observed due to poor base quality, poor mapping quality, nearness to a gap, strand bias in the observed variant and other bioinformatics artifacts. We use the final list of SNPs produced by such a procedure to identify blocks in the genome i.e. regions where 2 or more heterozygous SNPs fall within a 500 base region. For each block, we list all read pairs that overlap it and enumerate the local haplotype exhibited by each read pair (Figure 1). Thus starting from a set of filtered SNPs, this procedure examines the underlying read sequences to list a set of observed read-based haplotypes.

Next, we apply several data filters to minimize calling artifactual haplotypes. We ignore reads with mapping quality less than 30 and we ignore bases whose quality score is less than 30; quality score of 30 represents 1/1000 probability of error. We also ignore any read-based haplotypes that are supported by fewer than three reads.

**Figure 2:**
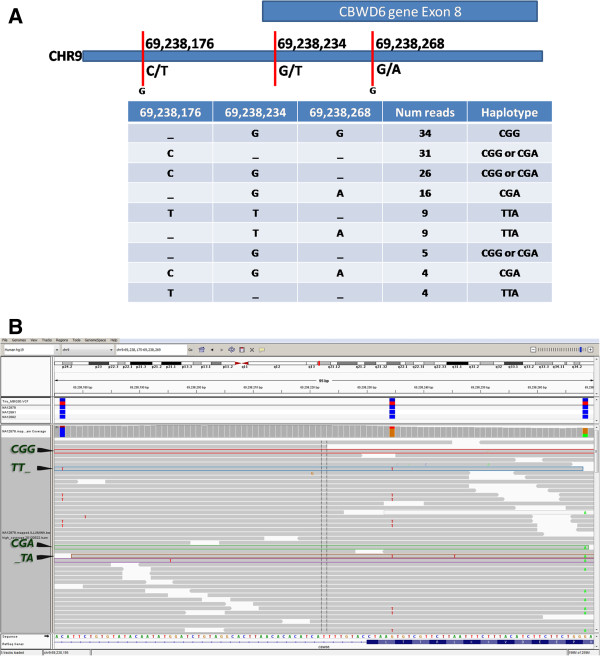
**A 3-SNP block is more complicated. A**. Cartoon of a 3-SNP block from CEU_TRIO member NA12878 on chromosome 9 overlapping exon 8 of gene CBWD6. The format is similar to Figure 1. **B**. Sequencing reads, as displayed on the Integrative Genomics Viewer [9] dramatically illustrate the fact that the “adjacent” SNPs in a block are not contiguous in the genome. Note the blanks in several read based haplotypes. Our parsimonious clustering procedure combines read-based haplotypes CG_ and _GA into CGA. Other possible completions of the CG_ partial haplotype (like CGT or CGC) are mathematically possible but excluded because they are not seen in the existing reads. It is clear that at least three haplotypes are required to support this data: CGG, CGA and TTA.

Thus, our haplotypes pass two sets of filters: the first set is included within our bioinformatics pipeline which filters out SNPs that are artifactual (e.g. have high strand bias or lie close to gaps) so that our blocks are based on filtered SNPs. The second set of filters reduces the set further by requiring high quality base calls, mapping and multiple observations of the same read based haplotype.When a block has more than 2 SNPs, read-based haplotypes might be partial i.e. cover only some of the SNPs. In these cases, we cluster the haplotypes to expand read-based haplotypes into local genomic haplotypes: if two read-based haplotypes overlap each other without contradicting, they can be clustered into a single longer haplotype (Figure 2). We call this procedure parsimonious clustering because it produces the minimum set of haplotypes required to explain the observed sequence data.

## Methods

We used the LHA procedure to identify the haplotypes in three different public data sets. All are from diploid organisms and hence, observation of more than two haplotypes is prima facie evidence that the tissue sequenced is heterogeneous. The three data sets are:

### CEU_Trio

A set of three whole genomes recommended [10] for benchmarking purposes belonging to a Central European (CEU) trio (NA12878, NA12891 and NA12892) from the Thousand Genomes project [11] were downloaded by FTP as aligned BAM files (approximately 71× coverage, mapped to HG19 version of the human genome) from the European Bioinformatics Institute [12]. Without re-mapping, we called variants in each sample as explained below.

### HNC_62

SRA files relating to 31 tumor and 31 matched normal (total 62 samples) tissues from patients with head and neck tumors [13] were downloaded from the Sequence Read Archive (SRA) [14]. We extracted fastq sequences from the SRA files in this exome sequencing data, and then used BWA [15] to map them to the HG19 reference human genome and to create a SAM alignment file. Next we used SAMtools [6] on each sample to generate a Binary Alignment and Mapping (BAM) file, to sort it and to remove Polymerase Chain Reaction (PCR) duplicates. We then used the Realigner-Target-Creator and Indel-Realigner modules of the GATK version 2.1.9 to refine alignments near all indels [7]. Finally, we called SNPs for all 62 samples as explained below.

### MUR_12

We downloaded the whole genome data for pure-bred laboratory strains of mouse [16]. We used the published BAM files (which mapped the reads to GRCm38_68 version of mouse genome) and called SNPs in the 12 strains.

### SNP calling

All SNP calling was done with the UnifiedGenotyper module of the GATK version 2.1.9 [7] using a minimum base quality threshold of 30 (‘-mbq 30’). The GATK caps the quality score of a base at its mapping quality and hence this also forces GATK to ignore any reads mapped with a quality less than 30. All samples in each data set were analyzed together but each data set was called separately. Thus, there were three separate runs of the UnifiedGenotyper for: (a) the three samples in CEU_TRIO, (b) 62 samples in HNC_62 and (c) and 12 samples in MUR_12.

### LHA procedure

Our program scanned the resulting Variant Call Format (VCF) files from each SNP-calling run to identify all blocks with 2 or 3 heterozygous SNPs within 500 bases of each other. Then, using the SAMtools application programming interface [17], our program read the BAM files to determine the base sequence at each SNP position for all reads overlapping any portion of the block. Reads that mapped with a quality score less than 30 are ignored. Likewise, if a read had a base with quality of less than 30 at a position, that read was considered to have skipped that position. Thus, we record in a file the high quality bases observed at each SNP position for every read mapping with high quality to any portion of the block.

Next we clustered together the read-based haplotypes for each block using the parsimony assumption i.e. if two read-based haplotypes overlapped without contradicting each other, they were combined into a possibly longer haplotype.

All called SNPs were annotated using Annovar [18] to determine their overlaps with genes, exons and segmental duplications.

## Results

### An illustrative example

Figure 1 shows a block of 2 SNPs from CEU_TRIO member NA12878. This block is on chromosome 3, overlapping the gene EPHA6. Given that the first SNP is heterozygous A/G and the second is G/T, there are four possible haplotypes i.e. *A*..*G*, *A*..*T*, *G*..*G* and *G*..*T*. (Theoretically, other haplotypes are also possible if a read has a base other than A or G at first SNP and/or other than G or T at the second. However, such instances are negligibly rare). If NA12878 were a uniform diploid genome, the data is expected to show two of these four haplotypes. However, examining the reads that span both these SNPs, we find evidence for three of the four haplotypes indicating that multiple cell types are present in the NA12878 sample.

It might be tempting to ignore the least populous haplotype i.e. to dismiss all the *A*..*T* reads as artifacts of erroneous mapping or sequencing. Note that, if this is done, the second SNP would not be called since only G would be seen mapped to that position. And the first SNP would also have lowered significance (and might not even be called) because of the removal of 7 out of 20 A’s from this position.

### 2-SNP blocks of CEU_TRIO

**Figure 3:**
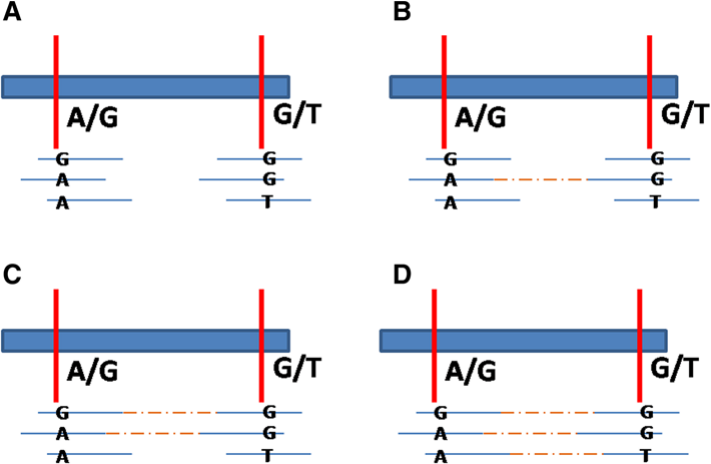
**Number of revealed haplotypes is often an under-estimate of the actual number of underlying haplotypes.** Four different hypothetical read mapping scenarios are shown for the block in Figure 1. All scenarios have same the number of reads at individual SNPs but differ in which of those reads span both SNPs. **(A)** 0-haplotype blocks. None of the reads spans the two SNPs therefore no haplotypes are deduced. **(B)** 1-haplotype blocks. A few reads span the two SNPs and they all show the same A.G haplotype. **(C, D)** 2- and 3-haplotype blocks arise when spanning reads show more haplotypes.

The top 3 rows of Table 1 show the number of haplotypes found in the 2-SNP blocks of CEU_TRIO. Each subject shows about 225,000 blocks with two SNPs each. Since both SNPs in the block were called heterozygous, each position individually has sufficient read coverage and shows two different bases mapped to it. However, depending on how the read (pairs) span the block, different number of haplotypes will be deduced (Figure 3) i.e. all underlying haplotypes are not always revealed. Two things are clear from the figure. First, barring sequencing or mapping error, the number of observed haplotypes is less than or equal to the actual number of underlying haplotypes but is never greater. Second, if the depth of coverage is increased, there is a greater likelihood of reads spanning both SNPs and revealing more underlying haplotypes.

**Table 1:** **Frequencies of haplotypes directly observed by LHA in 2-SNP blocks from the CEU_TRIO (whole genome) and HNC_62 (whole exome; aggregated into normal and tumor tissues)**

Subject	Total 2-blocks	Number of haplotypes in the block				
		0	1	2	3	4
NA12878*	225,139	86,719 (38%)	30,869 (14%)	103,339 (46%)	4,138 (2%)	73 (0%)
**NA12891**	224,079	75,756 (34%)	47,039 (21%)	96,263 (43%)	4,889 (2%)	132 (0%)
NA12892*	231,666	85,234 (37%)	50,738 (22%)	91,208 (39%)	4,394 (2%)	91 (0%)
**HNC-normal**	72,670	23,050 (32%)	8,535 (12%)	40,054 (55%)	1,006 (1.4%)	25 (0%)
**HNC-tumor**	73,591	23,100 (31%)	9,245 (13%)	40,005 (54%)	1,214 (1.6%)	27 (0%)

*NA12878 has one block with 6 haplotypes. NA12892 has one block with 5 haplotypes.

The largest proportion of the blocks (about 40%), show two haplotypes, complying with the expectation from a uniform diploid genomic sample. For about a third of the blocks no haplotypes can be deduced and in another 20% of the blocks reads spanning both SNPs show only one haplotype. Since both SNPs in the block were heterozygous we should always expect to see two haplotypes. Therefore these 0- and 1- haplotype blocks illustrate the fact that, for nearly half the blocks, read depth is not sufficient to reveal all underlying haplotypes using the conservative LHA procedure. So, with higher depth (than 71x), these blocks should show 2 haplotypes and some of them may show more.

About 2% of the blocks in each sample show 3 or more haplotypes. Since this data is from a normal human tissue with a diploid genome (at most 2 haplotypes expected), this observation of more than 2 haplotypes is prima facie evidence that the underlying genome is non-uniform or heterogeneous at these loci. Though it is only 2% of the blocks, this evidence cannot be ignored because (i) this still amounts to more than four thousand blocks (genomic loci) in each sample and (ii) this is a conservative estimate or lower bound of the number of loci showing heterogeneity.

### 2-SNP blocks for HNC_62 exome data

The last two rows of Table 1 show haplotype frequencies for HNC_62 whole exome data, aggregated for normal and tumor samples. Comparable to the number in CEU_TRIO, here also about 1.5% of the blocks display heterogeneity (have more than 2 haplotypes). These observations are also valid at a per-sample level (Additional file 1: Table S1).

### 2-SNP blocks for MUR_12

Presuming that inbred laboratory strains are homozygous, Keane et al. [16] analyzed MUR_12 genomes with a pipeline that assumed homozygosity. Specifically, they set prior probability of heterozygosity to be a hundred fold lower than the default with the result that they called very few heterozygous variants; their pipeline called about 6 million variants in each strain, of which only about 6 thousand were heterozygous (Additional file 2: Table S2). Using the authors’ variant list, no blocks were found i.e. heterozygous SNPs were rare enough that no two of them were within 500 bases of each other.

We independently re-analyzed Keane et al’s BAM files and called SNPs without requiring that all SNPs be homozygous, i.e. we used default value for the prior probability of heterozygosity. This resulted in up to 15% of the called SNPs being heterozygous (Additional file 2: Table S2).

Table 2 below shows that there were tens of thousands of 2-SNP blocks in each strain. About a quarter of all 2-SNP blocks show 2 haplotypes. If germlines of these purebred strains are expected to be homozygous they should have only one haplotype. Therefore in the case of these mice, even the 2 haplotype blocks raise the interesting question of whether the genome is in fact uniformly heterozygous instead of the mathematical possibility referred to earlier of two different cell lines with different genomes. Furthermore, 2-3% of 2-SNP blocks showed more than two haplotypes. Heterogeneity causing such haplotypes might arise from accumulation of replication errors during mitotic divisions and exposure to mutagens through the life of the organism [4]. Once again, the 0- and 1-haplotype blocks indicate that the depth of coverage (about 20× to 40×) in this study [16] was inadequate to enumerate all haplotypes present in the genome.

**Table 2:** **2-SNP blocks from 12 inbred mouse strains divided into blocks with 0, 1, 2, 3 or 4 different haplotypes directly observed using LHA**

Strain	Total 2-blocks	Number of haplotypes in the 2-SNP block				
		0	1	2	3	4
129P2	51,379	36,355 (70%)	13,420 (26%)	1,566 (3%)	37 (0%)	0 (0%)
AJ	76,834	27,027 (35%)	31,894 (42%)	16,346 (21%)	1,564 (2%)	3 (0%)
AKRJ	76,059	24,147 (31%)	29,400 (39%)	20,235 (27%)	2,263 (3%)	14 (0%)
BALBcJ	76,942	23,957 (31%)	30,319 (39%)	20,345 (26%)	2,319 (3%)	1 (0%)
C3HHeJ	83,505	26,814 (32%)	32,706 (39%)	21,555 (26%)	2,424 (3%)	6 (0%)
CASTEiJ	148,289	59,358 (40%)	54,330 (37%)	32,196 (22%)	2,393 (2%)	11 (0%)
CBAJ	81,455	27,571 (33%)	33,471 (41%)	18,562 (23%)	1,848 (2%)	3 (0%)
FVBNJ	75,529	23,653 (31%)	27,068 (36%)	21,885 (29%)	2,911 (4%)	12 (0%)
LPJ	80,790	28,320 (35%)	33,192 (41%)	17,521 (22%)	1,751 (2%)	6 (0%)
NODshiLtJ	80,305	27,229 (33%)	32,416 (40%)	18,621 (23%)	2,033 (3%)	6 (0%)
PWKPhJ	140,567	55,784 (40%)	51,437 (37%)	31,151 (22%)	2,182 (2%)	12 (0%)
SPRETEiJ	182,324	69,660 (38%)	62,565 (34%)	47,270 (26%)	2,804 (2%)	23 (0%)

### Blocks with 3 SNPs each

We did a similar analysis of 3-SNP blocks using the parsimonious clustering procedure (Figure 2) to determine the minimum number of haplotypes needed to explain the observed read pairs. With 3 SNPs up to eight haplotypes are possible in each block. (Theoretically, more than eight are possible if some reads show anomalous bases at one of the SNP positions. As noted before, such occurrences are negligibly rare). Table 3 shows the number of haplotypes directly observed at 3-SNP blocks for CEU_TRIO and for HNC_62 (aggregated into normal and tumor tissues).

**Table 3:** **Frequencies of haplotypes directly observed by LHA in 3-SNP blocks from the CEU_TRIO and HNC_62 (aggregated into normal and tumor tissues)**

Subject	Total 3-blocks	Number of haplotypes in the block								
		0	1	2	3	4	5	6	7	8
NA12878	108,968	12,090 (11.1%)	13,346 (12.2%)	78,392 (71.9%)	4,192 (3.8%)	752 (0.7%)	181 (0.2%)	14 (0%)	0 (0%)	1 (0%)
NA12891	107,888	13,278 (12.3%)	18,799 (17.4%)	70,454 (65.3%)	4,367 (4.0%)	789 (0.7%)	178 (0.2%)	20 (0%)	3 (0%)	0 (0%)
NA12892	111,699	16,198 (14.5%)	21,906 (19.6%)	68,915 (61.7%)	3,931 (3.5%)	613 (0.5%)	127 (0.1%)	9 (0%)	0 (0%)	0 (0%)
HNC-Normal	17,669	2,660 (15.1%)	2,025 (11.5%)	12,201 (69.1%)	521 (2.9%)	112 (0.6%)	115 (0.7%)	24 (0%)	11 (0%)	0 (0%)
HNC-Tumor	18,272	2,654 (14.5%)	2,239 (12.3%)	12,508 (68.5%)	573 (3.1%)	145 (0.8%)	122 (0.7%)	19 (0%)	12 (0%)	0 (0%)

In each sample, more than 4% of the 3-SNP blocks display 3 or more haplotypes, indicating directly observable heterogeneity. This pattern is preserved at a per sample level for HNC_62 (Additional file 3: Table S3).

The observation holds true for our re-analysis of MUR_12 genomes as well. We find tens of thousands of 3-SNP blocks and 3-5% of them show 3 or more haplotypes (Table 4). It is noteworthy here that the biggest proportion (more than 2/3 of the blocks) show 0 or 1 haplotype indicating that the 20× to 40× depth that Keane et al. report [16] is not sufficient to show all haplotypes present for 3-SNP blocks.

**Table 4:** **3-SNP blocks in MUR_12 genomes of inbred mouse strains divided into blocks with 0, 1, . 8 different haplotypes directly observed using LHA**

Strain	Total 3-blocks	Number of haplotypes in the block								
		0	1	2	3	4	5	6	7	8
129P2	17,486	11,472 (66%)	5,347 (31%)	635 (4%)	30 (0%)	0 (0%)	2 (0%)	0 (0%)	0 (0%)	0 (0%)
AJ	39,129	10,782 (28%)	18,585 (48%)	8,643 (22%)	1,061 (3%)	50 (0%)	7 (0%)	1 (0%)	0 (0%)	0 (0%)
AKRJ	40,220	9,677 (24%)	17,267 (43%)	11,507 (29%)	1,628 (4%)	122 (0%)	15 (0%)	3 (0%)	1 (0%)	0 (0%)
BALBcJ	40,865	9,750 (24%)	18,678 (46%)	10,823 (26%)	1,502 (4%)	99 (0%)	13 (0%)	0 (0%)	0 (0%)	0 (0%)
C3HHeJ	45,320	11,242 (25%)	20,750 (46%)	11,647 (26%)	1,592 (4%)	79 (0%)	9 (0%)	0 (0%)	1 (0%)	0 (0%)
CASTEiJ	75,328	23,462 (31%)	33,312 (44%)	17,014 (23%)	1,429 (2%)	83 (0%)	23 (0%)	3 (0%)	0 (0%)	2 (0%)
CBAJ	42,474	11,275 (26%)	20,102 (47%)	9,799 (23%)	1,236 (3%)	56 (0%)	4 (0%)	1 (0%)	1 (0%)	0 (0%)
FVBNJ	36,735	8,655 (24%)	15,234 (41%)	10,668 (29%)	1,909 (5%)	232 (1%)	31 (0%)	4 (0%)	0 (0%)	1 (0%)
LPJ	41,474	11,491 (28%)	19,605 (47%)	9,227 (22%)	1,065 (3%)	68 (0%)	15 (0%)	3 (0%)	0 (0%)	0 (0%)
NODshiLtJ	41,494	10,682 (26%)	19,240 (46%)	10,129 (24%)	1,317 (3%)	111 (0%)	11 (0%)	3 (0%)	0 (0%)	0 (0%)
PWKPhJ	70,702	22,248 (32%)	31,027 (44%)	15,825 (22%)	1,502 (2%)	72 (0%)	23 (0%)	4 (0%)	1 (0%)	0 (0%)
SPRETEiJ	95,263	25,115 (26%)	41,794 (44%)	26,270 (28%)	1,955 (2%)	109 (0%)	17 (0%)	2 (0%)	1 (0%)	0 (0%)

### Blocks with 4 or more SNPs each

In all three data sets, we also found blocks with 4 or more SNPS. However, analysis of such blocks is complicated by the presence of partial haplotypes (Figure 2) and generally lower mapping scores assigned to reads with multiple mismatches. We are formulating a statistical framework more robust than parsimonious clustering for properly analyzing such blocks.

### Where do the blocks occur?

Blocks can be classified into two categories: (i) Homogeneous blocks display 2 (or fewer haplotypes) and are not inconsistent with genomic homogeneity. (ii) Heterogeneous blocks display 3 (or more) haplotypes and are inconsistent with genomic homogeneity i.e. they cannot be explained without resorting to genomic heterogeneity.

**Figure 4:**
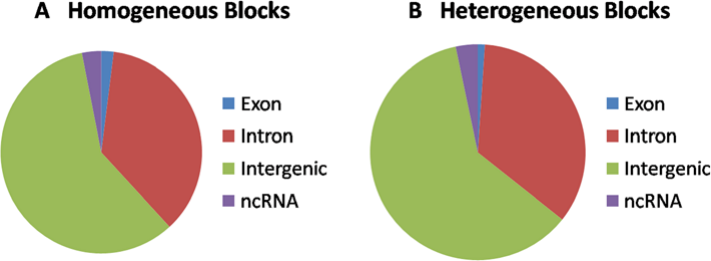
**Location of homogeneous (A) and heterogeneous (B) blocks, in relation to genes for the CEU_TRIO samples.** Note that both types of blocks occur in all types of locations (exonic, intronic and intergenic) in similar proportions.

**Figure 5:**
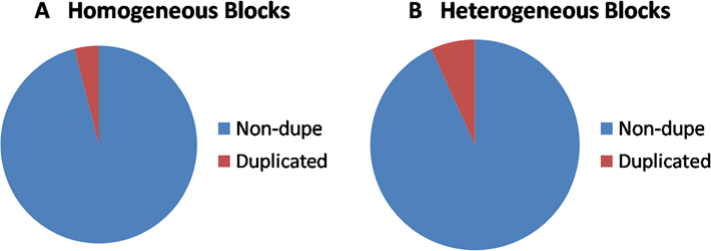
**Location of homogeneous (A) and heterogeneous (B) blocks in relation to known segmental duplications in the genome for CEU_TRIO samples.**

**Figure 6:**
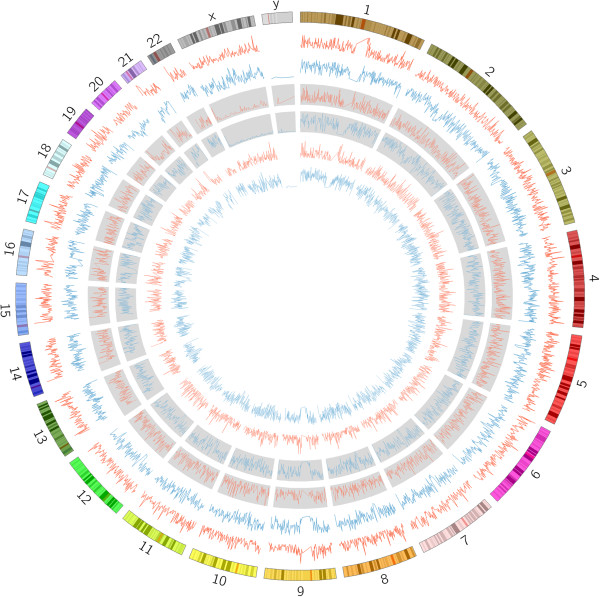
**Circos** [19] **plot of the density of blocks across the genome for the CEU_TRIO samples.** The blue plots represent density of homogeneous blocks and the red ones that of the heterogeneous blocks. The middle pair of plots (with the grey background) represents blocks from NA12891, the outer pair represents data from NA12878 and the inner pair is from NA12892.

Since artifactual mapping of reads can lead to enumerating three or more haplotypes, we analyzed if the blocks overlap regions that typically result in mapping errors. We annotated all our SNPS using Annovar [18] and analyzed if heterogeneous blocks occur in “non-functional” regions or in segmental duplications. Here, we present results only for CEU_TRIO though similar results are obtained for HNC_62 and MUR_12 as well. Figure 4B shows that heterogeneous blocks occur in all types of locations including exons, introns, intergenic regions and non-coding RNAs. Comparing to Figure 4A, which shows these proportions for homogeneous blocks, we notice that the proportions are similar even though homogeneous blocks appear slightly enriched for exonic locations.Figures 5A and B show that segmental duplications are more represented in the heterogeneous blocks but not overwhelmingly so. Indeed, 93% of heterogeneous blocks do not overlap any known segmental duplication.The greater representation for segmental duplications and non-exonic regions in the heterogeneous blocks might suggest that they result from mapping artifacts. However, the increase is marginal and a large number of heterogeneous blocks still remain even if we filter out blocks that overlap segmental duplications. Furthermore, our clustering procedure is parsimonious so that the number of haplotypes reported here is the lower bound on the real heterogeneity. Hence, many of the putatively homogeneous blocks (contributing to Figures 4A and 5A) might prove to be heterogeneous if there were greater depth of coverage resulting in more reads spanning multiple SNPs.Figure 6 plots the density of heterogeneous blocks for CEU_TRIO across the genome. As can be seen, heterogeneity hotspots are scattered all across the genome. It is notable that the number of homogeneous and heterogeneous blocks across the genome does not seem to follow any specific pattern. Therefore the observed heterogeneity cannot be discounted as caused by known hypervariable loci like immune genes.

## Discussion

We used local haplotyping analysis to examine sequencing reads that span 2 or 3 adjacent heterozygous SNPs. If the sequenced tissue has a uniform genome, sequencing reads in a block should only display two haplotypes in a diploid organism. Instead we found thousands of blocks where mapped sequencing read sets support three or more haplotypes. Evidence for heterogeneity of the underlying genome was directly observable in ordinary NGS data obtained from normal tissue of healthy individuals, as well as from normal and tumor tissues in patients with head and neck tumors.

Different blocks show different numbers of haplotypes. This is to be expected if different regions of the genome have different propensities for heterogeneity. Thus, LHA could provide a way to map heterogeneity hot spots in the genome. The nature and location of such hot spots might have important implications for predilection to disease. Also, it is noteworthy that this analysis was superimposed on SNPs that were already called in the traditional way. The bioinformatics procedures used for calling SNPs have their own assumptions (including that the underlying genome is diploid) which may unduly constrain regions marked as potentially heterogeneous. What is needed is a way to call haplotypes from raw NGS data without depending on called SNPs (manuscript in preparation).

LHA observed haplotypes are all local to within a block. We could consider global haplotypes at the whole genome level and ask: how many different genome-wide haplotypes exist in a sample? Mathematically, the block with the largest number of haplotypes provides a lower bound for whole genomic heterogeneity. Biologically, we must mitigate this estimate because of the possibility of sequencing or mapping errors and because some genes might be highly diverse i.e. not representative of overall genomic diversity.

It is important to consider at least two alternative explanations for observing 3 or more local haplotypes before we conclude that heterogeneity is real.

### Sequencing or mapping error?

The first alternate explanation involves sequencing or mapping error. In order to minimize this type of error, we instituted four filters: (i) we first called SNPs using the GATK UnifiedGenotyper so that we are only considering heterogeneity around SNPs that pass the thresholds for strand bias, nearness to gaps and other bioinformatics artifacts, (ii) we ignored sequence bases with Phred quality scores less than 30, (ii) we ignored all sequence reads with a mapping quality score less than 30, and (iv) we only considered read haplotypes supported by three or more reads (after the above filters were applied). Thus SNP calling software has passed these SNPs, the base-calling software has assigned less than 1/1000 probability of sequencing error and the mapping software has assigned less than 1/1000 probability of incorrect placement and we have at least three such observations for each read-based haplotype. As seen in Figure 2, the number of sequencing reads that must be ignored in order to assume a uniform diploid genome is often a large proportion of the mapped reads. Doing so, deletes variants called at many positions, calling into question many basic conclusions from a sequencing experiment.

Though it is formally not possible to address experimental error within the regime of the same experiment, these filters serve to remove the least confident portions of our results.

### Could it be due to segmental duplication?

It is possible that the regions of heterogeneity we are observing have multiple copies in the genome with subtle differences. In other words, the explanation could be that there are heterogeneous copies of a genomic locus rather than heterogeneity at a single locus. One way to examine this possibility is to see if heterogeneous blocks map mostly to known segmental duplications in the genome. We found that more than 90% of our heterogeneous blocks are outside of any regions known to be duplicated.

To throw more light on the duplication issue might need longer reads and/or much greater depth of coverage. Getting longer reads awaits technological improvements in sequencing. However, greater depth is feasible and we are currently in the middle of obtaining very deep sequencing. For this report, since more than 90% of the variants do not overlap known segmental duplication, this is unlikely to be the complete explanation for the observed heterogeneity.

#### Ways to experimentally validate heterogeneity

The most direct way to observe genome mosaicism is through single cell sequencing [20] of many different cells from the same tissue. Such technologies are still not broadly available in the market but preliminary results [21] suggest that genomic heterogeneity is real. Our analysis has shown that, even without the availability of single-cell sequencing technology, we can determine heterogeneity based on ordinary NGS data.

Another, somewhat indirect, way to validate heterogeneity is to see if similar conclusions are drawn when sequencing the same sample in a different technology. Recently Life Technology sequenced the exome of the CEU_TRIO using their Ion Torrent methodology and made this sequence available on their public server [22]. As partial validation we note that the heterogeneity of some of our exonic blocks is also observed in this data set (unpublished observations).

It is worth noting that Sanger sequencing, typically the “gold standard” for validating individual SNPs [23], is not likely to be useful for validating haplotypes. Even though Sanger reads are typically longer than NGS reads, they are averaged over a pool of genomic DNA from the tissue. Thus each SNP in the block will be seen as an ambiguous base and information about which bases at individual SNPs combine to form a haplotype is typically not forthcoming.

Given that single cell sequencing also appears [20, 21] to indicate heterogeneity in the normal genome, LHA-derived heterogeneity seems to have a basis in fact. Further its ability to determine heterogeneity from ordinary NGS data can be put to powerful use in analyzing existing data.

### Non-local haplotypes

Our procedure shows haplotypes at a local level in the genome. To observe similar combinations of SNPs that are far apart from each other might not be possible without single cell sequencing. However, statistical feature allocation methods [24] could indirectly infer such mosaic haplotypes over non-local SNPs or even SNPs on different chromosomes. One such method (Lee J, Muller P, Ji Y and Gulukota K, manuscript submitted) models haplotypes between non-local SNPs using a statistical technique called the Indian Buffet Process [25]. At one SNP, the alternate allele might be observed in 10% of the reads and in 75% of the reads at another. Our Indian Buffet Process analyzes such variable minor allele frequencies to assign SNPs to imputed subclones and to model possible global haplotypes.

## Conclusions

Local haplotyping analysis can provide directly observable evidence for heterogeneity and mosaicism using ordinary, though relatively deep, NGS data. Analyzing NGS data from three independent sources, we report that such heterogeneity is ubiquitous.

If genomes of normal tissues are heterogeneous at a large number of loci, the operational ramifications are quite dramatic. For example, the definition of cancer somatic mutations [26] might have to be altered because the germline is not uniquely defined. It might be important to periodically re-analyze a patient’s genome, if accumulation of replication errors over a life time leads to increased heterogeneity. Finally, in searching for genetic biomarkers, it might be important to consider not just genomic variants but also the heterogeneity context around them. New software will be needed for such analysis since existing software ignores this context.

## Electronic supplementary material

Additional file 1: Table S1: Lists the 31 subjects in the HNC_62 data set along with the frequencies of number of haplotypes observed in 2-SNP blocks of normal and tumor tissues. (XLS 22 KB)

Additional file 2: Table S2: Is an excel file and lists the number of SNPs called for each of the 12 strains in MUR_12 by our independent re-analysis. It also includes the same numbers from the VCF file derived from the original Keane et al. [16] analysis. (XLS 18 KB)

Additional file 3: Table S3: Is similar to Additional file 1: Table S1 except that it relates to 3 SNP blocks. (XLS 30 KB)

## Abbreviations

BAM: Binary SAM
CEU: Central European
GATK: Genome analysis tool kit
LHA: Local haplotyping analysis
NGS: Next generation sequencing
SAM: Sequence analysis and mapping
SNP: Single nucleotide polymorphism
SRA: Sequence read archive
VCF: Variant call format.
